# Prevalence of the *FMR1* Gene Premutation in Young Women with a Diminished Ovarian Reserve Included in an IVF Program: Implications for Clinical Practice

**DOI:** 10.3390/genes15081008

**Published:** 2024-08-01

**Authors:** Inés Agustí, Marta Méndez, Aina Borrás, Anna Goday, Marta Guimerà, Sara Peralta, Laura Ribera, Laia Rodriguez-Revenga, Dolors Manau

**Affiliations:** 1 Assisted Human Reproduction Unit, Gynecology Service, Clinic Institute of Gynecology, Obstetrics, and Neonatology (ICGON), Hospital Clínic Barcelona, 08036 Barcelona, Spain; iagustis@clinic.cat (I.A.); mamendez@clinic.cat (M.M.); aborras1@clinic.cat (A.B.); goday@clinic.cat (A.G.); mguimera@clinic.cat (M.G.); speralta@clinic.cat (S.P.); laribera@clinic.cat (L.R.); 2 Fundacio Clinic de Recerca Biomedique-Institut d’Investigacions Biomèdiques August Pi i Sunyer (IDIBAPS), 08036 Barcelona, Spain; 3 Biochemistry and Molecular Genetics Department, Hospital Clinic of Barcelona—Institut d’Investigacions Biomèdiques August Pi i Sunyer (IDIBAPS), 08036 Barcelona, Spain; lbodi@clinic.cat; 4 CIBER of Rare Diseases (CIBERER), Instituto de Salud Carlos III, 28029 Madrid, Spain

**Keywords:** *FMR1* premutation, premature ovarian failure, POSEIDON 3

## Abstract

The relationship between premature ovarian insufficiency (FXPOI) and premutation in the *FMR1* gene is well established. In recent years, though, a potential relationship between the latter and a low ovarian reserve has been suggested. To explore it, we conducted a retrospective study in an IVF program at a university tertiary referral center in Barcelona (Spain). Data were obtained retrospectively from a total of 385 women referred for *FMR1* gene testing at our institution from January 2018 to December 2021. We compared the prevalence of *FMR1* gene premutation between 93 of them, younger than 35 years, with a diminished ovarian reserve (DOR), characterized by levels of anti-Mullerian hormone < 1.1 ng/mL and antral follicle count < 5; and 132 egg donors screened by protocol that served as the controls. We found a higher prevalence of *FMR1* premutation in the DOR group (seven patients (7.69%)) than in the control group (one patient (1.32%)), Fisher-exact test *p*-value = 0.012). We concluded that compared with the general population represented by young egg donors, the prevalence of *FMR1* gene premutation is higher in young patients with a diminished ovarian reserve. Although these findings warrant further prospective validation in a larger cohort of patients within DOR, they suggest that, in clinical practice, *FMR1* premutation should be determined in infertile young patients with DOR in order to give them adequate genetic counselling.

## 1. Introduction

Fragile X syndrome (FXS) (#MIM300624; ORPHA 908) is the most common cause of inherited intellectual disability, affecting 1 in 4000 men and 1 in 8000 women [1]. It is caused by silencing of the *FMR1* gene (fragile X messenger ribonucleoprotein 1), due to the presence of a full mutation (>200 CGG repeats) in the 5’ untranslated region of the *FMR1* gene [2]. The *FMR1* gene is also the first single-gene cause of premature ovarian failure (fragile-X-associated premature ovarian insufficiency (FXPOI)) and one of the most common causes of ataxia (fragile-X-associated tremor/ataxia syndrome (FXTAS)). Both FXPOI and FXTAS are associated with the premutation of the *FMR1* gene (alleles within 55–200 CGG repeats) [3,4].

The association between *FMR1* gene premutation and premature ovarian insufficiency (POI) is well established [5]. Approximately 20% of women who carry *FMR1* gene premutation experience POI, whereas this occurs in only 1% of women in the general population [6]. Premature ovarian insufficiency (POI) is defined as the absence of menstruation (amenorrhea) due to the loss of ovarian function before the age of 40 years. Nevertheless, the guidelines of the European Society of Human Reproduction and Embryology (ESHRE) recommends the following, more specific, diagnostic criteria for POI: oligo/amenorrhea for at least four months and a follicle-stimulating hormone (FSH) level above 25 IU/l on two occasions separated by more than four weeks [7].

In the last decade, some studies have reported an association between the prevalence of *FMR1* premutation and a diminished ovarian reserve (DOR), but this is not well established because the results are controversial and mixed up by different *FMR1* repeat lengths [6,8,9,10,11].

The reception of donor oocytes is indicated for the treatment of ovarian failure. This assisted reproduction technique has high success rates, as indicated by high live birth rates. However, it entails relinquishing the gamete itself, which can be emotionally distressing for couples.

Recent reports from the World Health Organization reveal that 1 in 6 individuals will experience fertility issues throughout their lifetime [12]. This high prevalence is multifactorial, with a significant factor being the delay in women’s age at first pregnancy, especially in Western countries, particularly Southern Europe, where Spain has the lowest birth rate (according to the latest report from the Spanish Society of Fertility, it is estimated that 11% of all births in Spain are through assisted reproduction techniques [13]), only surpassed by Malta. This is particularly important because there is a physiological decline in fertility associated with the depletion in the number of oocytes, but also a decrease in the quality of oocytes with age. This leads to a reduction in spontaneous fertility but also impacts the success rates of assisted reproductive technologies (ART), where age influences the probability of achieving pregnancy [14].

During the initial assessment for in vitro fertilization (IVF), markers of the ovarian reserve are typically requested (including anti-Mullerian hormone (AMH) levels and the antral follicle count (AFC)). These markers have been established and standardized in the evaluation of patients with infertility. These markers guide the stimulation protocol required to achieve optimal ovarian stimulation.

Based on all of the above, in this study, we sought to determine the prevalence of *FMR1* premutation at an assisted human reproduction unit at a university, tertiary referral hospital. Specifically, we tested the hypothesis that *FMR1* premutation study should be determined in infertile young patients with DOR in order to give them adequate genetic counselling.

## 2. Methods

### 2.1. Study Population

Data from 385 women referred for *FMR1* gene testing at our institution from January 2018 to December 2021 were collected retrospectively. Clinic Barcelona is a university hospital that belongs to the Catalan Public Hospital Network. All women were visited at the Assisted Reproduction Unit, and molecular genetic testing was performed via the Biochemistry and Molecular Genetics Service, an accredited reference laboratory for these tests. Information on the demographic and clinical markers for ovarian reserve (AFC and AMH) was registered. An *FMR1* premutation is defined between 55 and 200 CGG repeats, while those below 55 are considered normal [15].

We reviewed the requests for this genetic testing and grouped these 385 women into 5 different categories: family history, premature ovarian insufficiency, young patients with a diminished ovarian reserve, oocyte donors, and miscellaneous.

From this data base, we selected the group with a diminished ovarian reserve (DOR), which included 93 women younger than 35 years. DOR was defined as those who presented regular cycles and altered ovarian reserved markers (AFC < 5, AMH < 1.1 ng/mL), in line with the POSEIDON criteria [16] that aim to identify patients with a poor prognosis in ART. The POSEIDON criteria consider the number of oocytes retrieved in previous stimulations, the age-related aneuploidy rate, and the ovarian “sensitivity” to exogenous gonadotropins, which impacts the total number of oocytes retrieved. These factors affect the final success rates of ART [17].

Additionally, we reviewed the various causes of infertility in the study group (such as male factor, tubal pathology or sterility of unknown origin among others).

As the control group, we used data from 132 cases of oocyte donors (who were screened for the *FMR1* gene by protocol).

### 2.2. Measurements

#### 2.2.1. CGG and AGG Determination

A peripheral venous blood sample was obtained in each woman. Genomic DNA was isolated by standard methods (Puregene and Purescript kits, Gentra, Minneapolis, MN, USA). The CGG repeat number and AGG interruptions were determined by a triplet repeat primed PCR (TP-PCR) using Asuragen AmplideX^®^ *FMR1* PCR Kit (Asuragen, Austin, TX, USA). Amplicons were evaluated on an ABI Prism 3130 Genetic Analyzer system (Applied Biosystems, Waltham, MA, USA), and GeneMapper software version 4.1 (Applied Biosystems) was used to determine the different sizes.

#### 2.2.2. Hormonal Determination

Biochemical parameters were measured in serum using standard methods in the Core Laboratory of our hospital, and the results were expressed according to female reference ranges. Anti-Mullerian hormone (AMH) was determined in the serum by a chemoluminescence immunoassay with paramagnetic particles for quantitative determination (AMH B13127 Beckman Coulter kit and in the ACCES2 device, Brea, CA, USA) (LOQ 0.02 ng/mL, interassay coefficient of variation < 5%; results expressed in ng/mL; normal female values between 1.0 and 4.5).

#### 2.2.3. Antral Follicle Count Determination

A Voluson S6 unit, General Electrics Medical Systems (Tiefenbach, Austria), equipped with a 5–7 MHz vaginal probe, was used. In this examination, a baseline gynecological assessment was performed to exclude gynecological pathology together with the antral follicle count (AFC). The AFC was calculated by measuring all the follicles with a diameter between 2 and 9 mm by ultrasound.

#### 2.2.4. Statistical Analysis

Continuous data are expressed as the mean and standard deviation, and categorical data as proportions. Normality was analyzed using the Shapiro–Wilk test. Groups were compared using Student’s *t*-test, the Mann–Whitney U test, the Chi-square test or the Fischer exact test, depending on data type and distribution. A *p*-value < 0.05 was considered statistically significant. Analyses were carried out with R (version 1.2).

## 3. Results

### 3.1. Study Population

**Figure 1:**
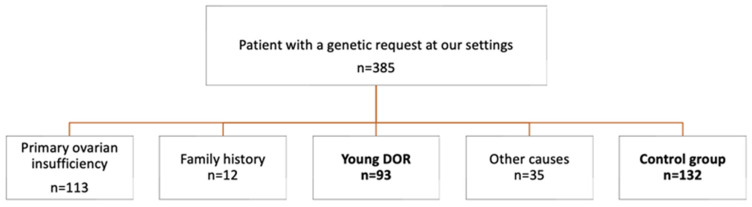
Consort diagram of the study. DOR: diminished ovarian reserve.

A total of 385 women referred for *FMR1* gene testing were examined between January 2018 and December 2021. The genetic testing indications were as follows (Figure 1): 113 (29.3%) patients with POI (established amenorrhea/oligomenorrhea and FSH > 25); 12 patients (3.1%) with a family history of the *FMR1* mutation; 93 patients (24.2%) were infertile normo-ovulatory and young (≤35 years old) women with DOR, defined as antral follicle count (AFC) < 5 and anti-Mullerian hormone < 1.1 ng/mL (according to POSEIDON criteria); and 35 patients (9.1%) for other causes, including consanguinity cases, non-sterile patients in other endocrinological studies, request errors, or patients older than 40 years. In the control group, we included 132 (34.3%) cases of oocyte donors, in whom *FMR1* gene testing was determined by protocol.

Regarding the study group, the causes of infertility were as follows: a total of 54 young DOR patients out of 93 (58.06%) came to our consultation due to infertility of unknown origin, followed by 14 (15.05%) due to a male cause; 11 patients from the total study population (11.83%) were diagnosed with endometriosis as a cause of sterility; and the tubal cause was present in 8 cases (8.60%). The rest of the patients (6 (6.45%)) attended for other reasons.

Figure 1 presents the consort diagram of the study and Table 1 the clinical characteristics of the study population. By focusing on the young DOR group (young patients who will undergo an IVF cycle), significant differences were observed in terms of age and ovarian reserve markers between the control group and the young DOR group. As expected, the young DOR group showed statistically significant decreased ovarian reserve markers, with low AMH levels (0.40 ± 0.29) and a lower AFC (4.9 ± 3.0) (Table 1).

**Figure 2:**
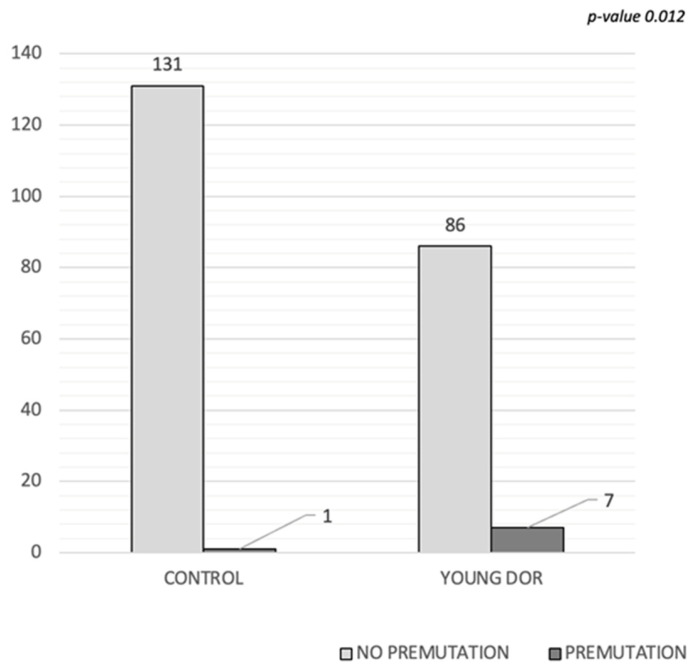
Fisher’s exact test *p* = 0.012. DOR: diminished ovarian reserve.

Figure 2 shows that seven patients carried the premutation in the young DOR group (7.69%), while only one patient (1.32%) did among the controls (Fisher’s exact test *p*-value = 0.012).

We also compared the levels of AMH, AFC, and the number of oocytes retrieved during ovarian stimulation between non-premutated and premutated patients in the study group. It was observed that the premutated patients in this group had lower AMH and AFC levels compared to the non-premutated patients (AMH 0.25 ng/mL vs. 0.4 ng/mL and AFC 4.4 vs. 5.1, respectively), and a reduced oocyte recovery as well (2.25 vs. 4.45 oocytes retrieved, respectively) (Table 2).

### 3.2. Treatment Received by the Young DOR Population

Regarding the treatments received by the young DOR population, 26 of these patients attempted one or more cycles of artificial insemination (27.9% of the cases). Of these 26, 9 ended up undergoing an IVF cycle and 4 achieved pregnancies. Nineteen patients attempted a natural-cycle IVF (20.4%), and six of them subsequently underwent one or more IVF cycles, and two pregnancies were achieved. Forty-one patients attempted a direct IVF cycle (44.1% of the population), achieving 13 pregnancies. A total of seven patients carried out an egg donation treatment at a private center.

Regarding the seven premutated patients, two underwent IVF pre-implantation genetic testing for monogenic disorders (PGT-M), three attempted IVF during a natural cycle, and four underwent egg donation.

The AGG interruptions in the premutation carriers were also assessed and no interruptions were found in any case, except one young DOR patient.

### 3.3. FMR1 Allele Size

Based on allelic groups, we created a table (Table 3) displaying their distribution within our population. In the control group, 131 individuals did not carry the premutation, while only 1 was detected with the premutation in the low repetition range. Conversely, within the young DOR group, seven patients were diagnosed with the premutation: four in the low repetition range, two in the medium range, and one with over 100 repetitions.

We looked at the occurrence of intermediate alleles (with 45 to 54 CGG repeats) independently. Among the study group, two exhibited an intermediate range of CGG repeats. We compared the intermediate range of triples between both groups, but found no differences (*p*-value of 0.30).

We also reviewed the ovarian reserve markers in two subgroups of the non-premutation patient group. In the low-repeat group (<26 CGG repeats on both alleles), 7 women out of 84 were identified with a mean AMH level of 0.84 ng/mL and a mean AFC of 6.3. Additionally, there were two individuals in the intermediate (or gray) zone with a mean AMH level of 0.7 ng/mL and an AFC of 5. These findings are consistent with the expectations for the group characterized by a low ovarian reserve in young patients (POSEIDON Group 3). Unfortunately, the current sample size is insufficient to perform statistical tests and draw definitive conclusions from these two subgroups.

## 4. Discussion

This study shows that the prevalence of premutation of the *FMR1* gene in women younger than 35 years of age with infertility and low ovarian reserve markers according to POSEIDON criteria was higher ((7.69%) than in the controls (oocyte donors; 1.32%, *p* = 0.012)).

Approximately 1/250 females and 1/800 males carry premutation alleles in the range of 55–200 repeats. Among women who carry the premutation, 20–24% have POI, compared with only 1% in the general population [18]. Overall, premutation carriers go through menopause ~5 years earlier than non-carriers and, among those still cycling, have higher FSH levels [19,20,21].

The molecular basis of FXPOI has been poorly studied, and we still do not fully understand the role that the *FMR1* gene plays in ovarian function, or the molecular pathways involved in FXPOI.

Another important question is why ovarian dysfunction is limited only to women carrying the premutation. Several theories have been proposed to explain this, with one of the most accepted being that the messenger RNA (mRNA) levels of the *FMR1* gene in carriers are increased [22], leading to a gain-of-function of this mRNA, with toxic effects on the cell [23]. Another theory is that the expanded CGG repeat tract (expCGG mRNA) could be interfering with normal follicle development [24,25,26]. It has been shown that expCGG mRNA is capable of sequestering proteins and other transcripts, forming intranuclear inclusions detected in ovarian stromal cells and other tissues [27]. These inclusions contain toxic polypeptides (FMRpolyG), which, together with the inclusions and increased levels of expCGG mRNA, contribute to the development of various diseases associated with the premutation of the gene: FXPOI and FXTAS [28].

The association between the CGG repeat length in the *FMR1* gene and POI risk has also been a topic of interest, but the results from individual studies were inconsistent and sometimes contradictory, especially for idiopathic POI. Four allelic groups have been distinguished based on the number of *FMR1* CGG repeats: normal alleles range from 5 to 44 repeats; intermediate alleles (also called grey zone) span 44 to 54 repetitions; premutation alleles range from 55 to 200 repeats; and full mutations consist of over 200 repeats [15]. Intermediate and premutation alleles can be unstable when transmitted from the mother to the child, leading to a full mutation across several generations.

Regarding the premutation allelic group (55 to 200 repetitions), we can distinguish three subgroups: low number with repetitions from 55 to 79 reps., medium number with 80–99 reps., and high number with more than 100 reps. Interestingly, the association of the repeat size with the risk is non-linear. In 2007, a study showed a non-linear relationship among premutation carriers and ovarian insufficiency [18]. The mid-range repeat size group (80–99 repeats), not the highest group, had an increased risk for altered cycle traits (shortened cycle length, irregular cycles and skipped cycles), subfertility, and dizygotic twinning [18]. In our study, four patients in the young DOR group had premutation within the low range of CGG repeats and two were within the intermediate range. Only one was classified within the high range. We compared the intermediate range of triples between both groups, but found no differences (*p*-value of 0.30).

Although it is well known that *FMR1* premutation is associated with POI, the prevalence of premutation in a population of patients with a low ovarian reserve and its implications in routine clinical practice is unknown. Previous studies have demonstrated that the prevalence of *FMR1* premutation is increased in women with DOR compared with women with other causes of infertility and oocyte donors [6]. Other studies have suggested that the premutation, as well as the intermediate repeat length and high normal repeat length (35–44 repeats) are associated with POI. But the literature is inconsistent on the association between the *FMR1* trinucleotide repeat length and infertility [29,30,31,32,33]. However, in a population-based study, Murray et al. [33] revealed that premutation-sized *FMR1* repeats are substantial risk factors for POI, whereas intermediate alleles were not. Other studies reported a significant increase in the number of intermediate and premutation *FMR1* alleles in DOR patients compared with the controls [6,8,31,34]. On the other hand, De Geyter et al. [35], in their prospective cohort study, found that neither of the categories of *FMR1* CGG repeat length expansions (premutation, intermediate range) was more prevalent in infertile women with POI than control women, nor was the CGG repeat length correlated with the severity of premature ovarian insufficiency. In 2009, Streuli et al. published a retrospective study in which a group of women with infertility and BRO was compared with one of infertile women with a normal ovarian reserve (control). Their results suggested that there was a greater risk in the group of BRO women of presenting a number of repetitions in the intermediate or premutated range (>40 CGG repetitions) compared to the control group [8]. Pastore et al. have published extensively on this topic. In 2014, they reported that there appeared to be a higher rate of follicular loss beginning at older ages in women with BRO and ≥35 CGG repeats [36]. Subsequently, in 2017, they investigated whether associations between CGG repeat lengths differed between women diagnosed with BRO from population controls, and whether associations varied by racial/ethnic group. No significant differences were found in the normal/intermediate range between the cases and controls or by racial/ethnic group, and they concluded that the study rejected the association between BRO and high/normal/intermediate repetitions, and confirmed an association between BRO and low/normal repetitions in white women [10]. That same year, Man et al., in their review, proposed a new concept: a fragile-X-associated diminished ovarian reserve (FXDOR). This diagnosis will be a diagnosis of exclusion, after excluding all other known reasons for infertility (for instance, the male factor, endometriosis, mechanical factor, etc.) in a woman carrying a premutation allele with regular menstruations, regardless of the levels of ovarian markers, younger than 40 years of age. They also reported that there was a higher risk of FXPOI if the woman had between 80 and 100 CGG repetitions vs. >100 CGG repetitions. They proposed a phenotype in women with a premutation, which derived from the combination of different damages that occur in stages of development and maintenance of the follicular pool [37]. Eslami et al. recently published that the frequency of *FMR1* premutation in both DOR and POI patients was significantly higher than that in the control group, and presented high FSH levels and low AMH levels. Moreover, they concluded that intermediate-sized *FMR1* CGG repeat alleles should not be considered a high risk factor for POI and DOR [9]. These data are supported by a recently published meta-analysis that reported on the association between CGG repeat lengths of the *FMR1* gene and the severity of POI. Thirteen studies, comprising 2047 cases and 6912 controls, were included to assess premutation and intermediate repeat length in POI patients. Premutation of the *FMR1* gene showed a significant association with POI compared to the controls, while there was no significant correlation found between intermediate repeat length and POI. Additionally, six studies, involving 975 patients and 1749 controls, were eligible for evaluating premutation and intermediate repeat length in DOR. Premutation was significantly associated with DOR, whereas no significant correlation was found between an intermediate repeat length and DOR in the case–control comparison [5].

It is also important to consider the number of AGG interruptions. These trinucleotides typically appear at the 5′ end of the CGG repeat sequence [38]. They are described within the *FMR1* gene as a marker contributing to gene stability and potentially associated with the development of FXPOI, thereby implicating ovarian dysfunction in the process. Several studies have reported that premutation carriers, particularly those with at least one AGG interruption within the CGG repeat sequence, are less prone to experiencing an expansion of the CGG repeat length into a full mutation. Consequently, they are less likely to have a child with fragile X syndrome (FXS) in the following generation [39,40,41,42]. Lekovic et al. demonstrated a direct correlation between the number of AGG interruptions and ovarian reserve parameters a few years ago. Their findings indicated that patients with longer uninterrupted CGG repeats following AGG interruptions exhibited the lowest ovarian reserve [11]. In our assessment of AGG interruptions among premutation carriers, no interruptions were detected in any case, except for one young DOR patient.

There are few published studies on the outcomes of ART in these patients. Generally, a decreased response to ovarian stimulation has been suggested [43,44]. In 2010, a study was published to determine if there was a correlation between the number of CGG repeats and the number of retrieved oocytes, concluding that women with a number of repeats between 80 and 120 responded with fewer oocytes [45]. On the contrary, Geyter et al. found no correlation between the number of CGG repeats and markers of ovarian reserve parameters. Therefore, they concluded that infertility (even with a reduced ovarian reserve) is not suitable to identify the possible premutated patients [35]. Similarly, Friedman-Gohas also observed no associations between the number of CGG repeats or AGG interruptions and any of the controlled ovarian hyperstimulation variables [28]. Avraham et al., in their 2017 retrospective study, analyzed 309 fresh cycles overall (of 21 patients with a full mutation of the *FMR1* gene and 51 premutation carriers). The results suggested that the premutation carriers displayed a reduced ovarian response (fewer oocytes retrieved) and full-mutation patients had a normal response. There was no significant difference between premutation carriers and full-mutation patients with regards to the fertilization rate, cleavage rate or biopsy rate [43]. Elizur’s group reported that this reduction in ovarian response to stimulation could be due to the accumulation of *FMR1* mRNA in ovarian granulosa cells [24]. In a review conducted by the Pastore group in 2019, they explored the outcomes of ART in relation to the premutation status of the *FMR1* gene, reporting that women with the premutation had a decreased pregnancy rate due to a lower number of retrieved oocytes compared to women without the premutation or with a full mutation [46]. In 2023, in a Chinese study pending publication, the researchers investigated whether CGG repeats affected the ovarian function or response during the IVF cycle, observing a negative correlation between CGG repeats and serum AMH, E2, AFC, and oocyte yield, but no significant differences were found between CGG repeats and the embryo quality or live birth rate [47].

On the other hand, different studies set 35 years as the cut-off age because from this age onwards, the ovarian reserve decreases markedly and the alteration in the ovarian reserve markers can be explained by the aging process of the woman [9,48].

Additionally, the use of POSEIDON criteria has allowed for not only optimizing the IVF cycle in a specific patient, but also guiding clinicians in assessing the need for complementary tests (including genetic), thus personalizing the treatment during a medical consultation. Along this line, our study has tried to find a patient profile that clusters the characteristics (POSEIDON Group 3) in order to assess the status of the fragile X gene, since it will modify the prognosis and the future of the patient. According to the latest literature, the patient population belonging to POSEIDON Group 3 constitutes approximately 10% of the POSEIDON population [16,49]. Based on these data, the prevalence of POSEIDON 3 patients in our center is 13%, with genetic testing for fragile X being conducted in 80% of these patients.

The diagnosis of a premutation implies several consequences in different areas such as at the reproductive level, because the patient with the premutation will be reoriented to IVF with an implantation genetic diagnosis in order to avoid the development of FXS in the offspring. The failure to diagnose it could lead to affected children, with a high economic and social impact. However, it is important to remember that professionals and patients should be aware of the limitations of this procedure which, on one hand, are due to the quantity of fertile oocytes (which directly depends on the patient’s response to stimulation), and on the other hand, the laboratory technique itself [50].

The American College of Medical Genetics and the American College of Obstetrics and Gynecology guidelines in 2010 [51] recommended the evaluation of women with infertility and/or elevated FSH for the *FMR1* premutation based on the phenotype in known carriers [18,19,20,52,53]. In 2008, Rajendra et al. [54] reported that half of newly diagnosed fragile X families were discovered due to the birth of an affected child. This reinforces the idea of studying women with DOR before having an affected child in the same generation (for premutation carriers). Currently, neither the European nor the American assisted human reproduction guidelines, in contrast to the two mentioned above, recommend screening for the fragile X gene in patients with low ovarian reserve markers.

Regarding the screening protocol for oocyte donors, the Spanish Fertility Society and the American Society for Reproductive Medicine have endorsed genetic testing for the *FMR1* gene in women willing to donate their eggs [55,56]. However, the European Society of Fertility keeps the option to request such a study open [57]. Regarding the Australian and New Zealand Fertility Society, relevant genetic studies are recommended according to the donor’s ethnicity (https://www.fertilitysociety.com.au/donor-programme-australia-new-zealand/#screening-egg, accessed on 29 July 2024). Finally, the Canadian Fertility Society recommends genetic testing if a disease is suspected after completing a structured questionnaire (https://www.canada.ca/en/health-canada/services/publications/drugs-health-products/technical-directive-sperm-ova-donors.html#a2.1.2, accessed on 29 July 2024).

Several strengths of our study are worth mentioning. First, it is the first study to investigate the prevalence of *FMR1* gene premutation among young patients with a diminished ovarian reserve undergoing IVF treatment, finding a higher prevalence of premutation. Additionally, regarding the prevalence of the premutation in young women with a diminished ovarian reserve, there is little published on this topic, and the results are inconsistent [6,9,11,31] and with little clinical translation. Moreover, in our clinical practice, young patients with DOR are frequent and they demand to continue with IVF cycles and have biologically related offspring. We consider that this group could be a good profile of patients eligible to perform the genetic study. On the other hand, we acknowledge several potential limitations, including that our study is a retrospective review of *FMR1* gene results, with limited determinations of *FMR1* studies, and that donor screening and young infertile patients with a significantly low ovarian reserve were the main indications to request the *FMR1* status gene, which might have led to selection bias.

## 5. Conclusions

We concluded that the prevalence of *FMR1* premutation is significantly higher in young women with DOR than in the general population. These results should be confirmed prospectively in a larger population of POSEIDON 3 patients to provide them adequate clinical and genetic counselling to minimize the impact and consequences that could arise from a late diagnosis.

## Figures and Tables

**Table 1:** Clinical characteristics of the women included in the study.

PATIENT GROUP	N	AGE	AMH	AFC
YOUNG DOR	93	31.813 ± 2.551	0.406 ± 0.291	4.988 ± 3.058
CONTROL	132	25.705 ± 0.4.704	3.073 ± 0.993	21.724 ± 9.726

AMH: anti-Mullerian hormone levels; AFC: antral follicular count.

**Table 2:** Comparison between premutated and non-premutated patients in the study group.

	AMH (ng/mL) (Mean)	AFC (Mean)	Number of Oocytes (Mean)
No premutation DOR (*n* = 86)	0.4	5.1	4.45
Premutation DOR (*n* = 7)	0.26	4.4	2.25

AMH: anti-Mullerian hormone levels; AFC: antral follicular count.

**Table 3:** Frequency of different *FMR1* repeat sizes among the study groups. Fisher’s exact test *p* = 0.3032.

	NON-CARRIER		PREMUTATION		
Group	COMMON < 45	INTERMEDIATE 45–54	LOW 55–79	MEDIUM 80–99	HIGH > 100
CONTROL	131 (99.24%)	-	1 (0.76%)	-	-
YOUNG DOR	84 (90.32%)	2 (2.15%)	4 (4.30%)	2 (2.15%)	1 (1.08%)

## Data Availability

The original contributions presented in the study are included in the article, further inquiries can be directed to the corresponding author.

## References

[1] Turner G., Webb T., Wake S., Robinson H. (1996). Prevalence of fragile X syndrome. Am. J. Med. Genet..

[2] Fu Y.H., Kuhl D.P., Pizzuti A., Pieretti M., Sutcliffe J.S., Richards S., Verkerk A.J., Holden J.J., Fenwick R.G., Warren S.T. (1991). Variation of the CGG repeat at the fragile X site results in genetic instability: Resolution of the Sherman paradox. Cell.

[3] Cronister A., Schreiner R., Wittenberger M., Amiri K., Harris K., Hagerman R.J. (1991). Heterozygous fragile X female: Historical, physical, cognitive, and cytogenetic features. Am. J. Med. Genet..

[4] Hagerman R.J., Amiri K., Cronister A. (1991). Fragile X checklist. Am. J. Med. Genet..

[5] Huang J., Zhang W., Liu Y., Liu Y., Wang J., Jiang H. (2019). Association between the FMR1 CGG repeat lengths and the severity of idiopathic primary ovarian insufficiency: A meta analysis. Artif. Cells Nanomed. Biotechnol..

[6] Karimov C.B., Moragianni V.A., Cronister A., Srouji S., Petrozza J., Racowsky C., Ginsburg E., Thornton K.L., Welt C.K. (2011). Increased frequency of occult fragile X-associated primary ovarian insufficiency in infertile women with evidence of impaired ovarian function. Hum. Reprod..

[7] European Society of Human Reproduction and Embryology, (ESHRE) Evidence-Based Guideline: Premature Ovarian Insufficiency. 2024, Draft for Review. https://www.google.com/url?sa=t&source=web&rct=j&opi=89978449&url=https://www.eshre.eu/-/media/sitecore-files/Guidelines/POI/2024/ESHRE-GUIDELINE_POI_DRAFT-FOR-REVIEW_2024.pdf&ved=2ahUKEwj1u8WJydCHAxWQzQIHHSBbELsQFnoECBgQAQ&usg=AOvVaw1PpYJ3qMrOFGvRf3ONVaKT.

[8] Streuli I., Fraisse T., Ibecheole V., Moix I., Morris M.A., de Ziegler D. (2009). Intermediate and premutation FMR1 alleles in women with occult primary ovarian insufficiency. Fertil. Steril..

[9] Eslami A., Farahmand K., Totonchi M., Madani T., Asadpour U., Zari Moradi S., Gourabi H., Mohseni-Meybodi A. (2017). FMR1 premutation: Not only important in premature ovarian failure but also in diminished ovarian reserve. Hum. Fertil..

[10] Pastore L.M., Young S.L., Manichaikul A., Baker V.L., Wang X.Q., Finkelstein J.S. (2017). Distribution of the FMR1 gene in females by race/ethnicity: Women with diminished ovarian reserve versus women with normal fertility (SWAN study). Fertil. Steril..

[11] Lekovich J., Man L., Xu K., Canon C., Lilienthal D., Stewart J.D., Pereira N., Rosenwaks Z., Gerhardt J. (2018). CGG repeat length and AGG interruptions as indicators of fragile X-associated diminished ovarian reserve. Genet. Med..

[12] World Health Organization (2023). Infertility Prevalence Estimates.

[13] Sociedad Española de Fertilidad (2021). Registro Nacional de Actividad 2021. Registro SEF. https://www.registrosef.com/public/docs/sef2021_IAFIV.pdf.

[14] Franasiak J.M., Forman E.J., Hong K.H., Werner M.D., Upham K.M., Treff N.R., Scott R.T. (2014). The nature of aneuploidy with increasing age of the female partner: A review of 15,169 consecutive trophectoderm biopsies evaluated with comprehensive chromosomal screening. Fertil. Steril..

[15] Asadi R., Omrani M.D., Ghaedi H., Mirfakhraie R., Azargashb E., Habibi M., Pouresmaeili F. (2018). Premutations of FMR1 CGG repeats are not related to idiopathic premature ovarian failure in Iranian patients: A case control study. Gene.

[16] Drakopoulos P., Bardhi E., Boudry L., Vaiarelli A., Makrigiannakis A., Esteves S.C., Tournaye H., Blockeel C. (2020). Update on the management of poor ovarian response in IVF: The shift from Bologna criteria to the Poseidon concept. Ther. Adv. Reprod. Health.

[17] Esteves S.C., Yarali H., Vuong L.N., Conforti A., Humaidan P., Alviggi C. (2022). POSEIDON groups and their distinct reproductive outcomes: Effectiveness and cost-effectiveness insights from real-world data research. Best Pract. Res. Clin. Obstet. Gynaecol..

[18] Allen E.G., Sullivan A.K., Marcus M., Small C., Dominguez C., Epstein M.P., Charen K., He W., Taylor K.C., Sherman S.L. (2007). Examination of reproductive aging milestones among women who carry the FMR1 premutation. Hum. Reprod..

[19] Hundscheid R.D., Braat D.D., Kiemeney L.A., Smits A.P., Thomas C.M. (2001). Increased serum FSH in female fragile X premutation carriers with either regular menstrual cycles or on oral contraceptives. Hum. Reprod..

[20] Murray A. (2000). Premature ovarian failure and the FMR1 gene. Semin. Reprod. Med..

[21] Sullivan A.K., Marcus M., Epstein M.P., Allen E.G., Anido A.E., Paquin J.J., Yadav-Shah M., Sherman S.L. (2005). Association of FMR1 repeat size with ovarian dysfunction. Hum. Reprod..

[22] Tassone F., Hagerman R.J., Taylor A.K., Mills J.B., Harris S.W., Gane L.W., Hagerman P.J. (2000). Clinical involvement and protein expression in individuals with the FMR1 premutation. Am. J. Med. Genet..

[23] Mila M., Alvarez-Mora M.I., Madrigal I., Rodriguez-Revenga L. (2018). Fragile X syndrome: An overview and update of the FMR1 gene. Clin. Genet..

[24] Elizur S.E., Lebovitz O., Derech-Haim S., Dratviman-Storobinsky O., Feldman B., Dor J., Orvieto R., Cohen Y. (2014). Elevated levels of FMR1 mRNA in granulosa cells are associated with low ovarian reserve in FMR1 premutation carriers. PLoS ONE.

[25] Sherman S.L., Curnow E.C., Easley C.A., Jin P., Hukema R.K., Tejada M.I., Willemsen R., Usdin K. (2014). Use of model systems to understand the etiology of fragile X-associated primary ovarian insufficiency (FXPOI). J. Neurodev. Disord..

[26] Hagerman P. (2013). Fragile X-associated tremor/ataxia syndrome (FXTAS): Pathology and mechanisms. Acta Neuropathol..

[27] Buijsen R.A., Visser J.A., Kramer P., Severijnen E.A., Gearing M., Charlet-Berguerand N., Sherman S.L., Berman R.F., Willemsen R., Hukema R.K. (2016). Presence of inclusions positive for polyglycine containing protein, FMRpolyG, indicates that repeat-associated non-AUG translation plays a role in fragile X-associated primary ovarian insufficiency. Hum. Reprod..

[28] Friedman-Gohas M., Elizur S.E., Dratviman-Storobinsky O., Aizer A., Haas J., Raanani H., Orvieto R., Cohen Y. (2020). FMRpolyG accumulates in FMR1 premutation granulosa cells. J. Ovarian Res..

[29] Bretherick K.L., Fluker M.R., Robinson W.P. (2005). FMR1 repeat sizes in the gray zone and high end of the normal range are associated with premature ovarian failure. Hum. Genet..

[30] Bodega B., Bione S., Dalpra L., Toniolo D., Ornaghi F., Vegetti W., Ginelli E., Marozzi A. (2006). Influence of intermediate and uninterrupted FMR1 CGG expansions in premature ovarian failure manifestation. Hum. Reprod..

[31] Gleicher N., Weghofer A., Barad D.H. (2009). A pilot study of premature ovarian senescence: I. Correlation of triple CGG repeats on the FMR1 gene to ovarian reserve parameters FSH and anti-Mullerian hormone. Fertil. Steril..

[32] Bennett C.E., Conway G.S., Macpherson J.N., Jacobs P.A., Murray A. (2010). Intermediate sized CGG repeats are not a common cause of idiopathic premature ovarian failure. Hum. Reprod..

[33] Murray A., Schoemaker M.J., Bennett C.E., Ennis S., Macpherson J.N., Jones M., Morris D.H., Orr N., Ashworth A., Jacobs P.A. (2014). Population-based estimates of the prevalence of FMR1 expansion mutations in women with early menopause and primary ovarian insufficiency. Genet. Med..

[34] Barasoain M., Barrenetxea G., Huerta I., Telez M., Carrillo A., Perez C., Criado B., Arrieta I. (2013). Study of FMR1 gene association with ovarian dysfunction in a sample from the Basque Country. Gene.

[35] De Geyter C., M’Rabet N., De Geyter J., Zurcher S., Moffat R., Bosch N., Zhang H., Heinimann K. (2014). Similar prevalence of expanded CGG repeat lengths in the fragile X mental retardation I gene among infertile women and among women with proven fertility: A prospective study. Genet. Med..

[36] Pastore L.M., McMurry T.L., Williams C.D., Baker V.L., Young S.L. (2014). AMH in women with diminished ovarian reserve: Potential differences by FMR1 CGG repeat level. J. Assist. Reprod. Genet..

[37] Man L., Lekovich J., Rosenwaks Z., Gerhardt J. (2017). Fragile X-Associated Diminished Ovarian Reserve and Primary Ovarian Insufficiency from Molecular Mechanisms to Clinical Manifestations. Front. Mol. Neurosci..

[38] Kunst C.B., Leeflang E.P., Iber J.C., Arnheim N., Warren S.T. (1997). The effect of FMR1 CGG repeat interruptions on mutation frequency as measured by sperm typing. J. Med. Genet..

[39] Nolin S.L., Glicksman A., Ersalesi N., Dobkin C., Brown W.T., Cao R., Blatt E., Sah S., Latham G.J., Hadd A.G. (2015). Fragile X full mutation expansions are inhibited by one or more AGG interruptions in premutation carriers. Genet. Med..

[40] Eichler E.E., Holden J.J., Popovich B.W., Reiss A.L., Snow K., Thibodeau S.N., Richards C.S., Ward P.A., Nelson D.L. (1994). Length of uninterrupted CGG repeats determines instability in the FMR1 gene. Nat. Genet..

[41] Yrigollen C.M., Durbin-Johnson B., Gane L., Nelson D.L., Hagerman R., Hagerman P.J., Tassone F. (2012). AGG interruptions within the maternal FMR1 gene reduce the risk of offspring with fragile X syndrome. Genet. Med..

[42] Nolin S.L., Sah S., Glicksman A., Sherman S.L., Allen E., Berry-Kravis E., Tassone F., Yrigollen C., Cronister A., Jodah M. (2013). Fragile X AGG analysis provides new risk predictions for 45-69 repeat alleles. Am. J. Med. Genet. A.

[43] Avraham S., Almog B., Reches A., Zakar L., Malcov M., Sokolov A., Alpern S., Azem F. (2017). The ovarian response in fragile X patients and premutation carriers undergoing IVF-PGD: Reappraisal. Hum. Reprod..

[44] La Marca A., Mastellari E. (2021). Fertility preservation for genetic diseases leading to premature ovarian insufficiency (POI). J. Assist. Reprod. Genet..

[45] Bibi G., Malcov M., Yuval Y., Reches A., Ben-Yosef D., Almog B., Amit A., Azem F. (2010). The effect of CGG repeat number on ovarian response among fragile X premutation carriers undergoing preimplantation genetic diagnosis. Fertil. Steril..

[46] Pastore L.M., Christianson M.S., McGuinness B., Vaught K.C., Maher J.Y., Kearns W.G. (2019). Does theFMR1 gene affect IVF success?. Reprod. BioMedicine Online.

[47] Jin X., Zeng W., Xu Y., Jin P., Dong M. (2023). CGG repeats of FMR1 negatively affect ovarian reserve and response in Chinese women. Reprod. BioMedicine Online.

[48] Pastore L.M., Johnson J. (2014). The FMR1 gene, infertility, and reproductive decision-making: A review. Front. Genet..

[49] Haahr T., Dosouto C., Alviggi C., Esteves S.C., Humaidan P. (2019). Management Strategies for POSEIDON Groups 3 and 4. Front. Endocri..

[50] Persico T., Tranquillo M.L., Seracchioli R., Zuccarello D., Sorrentino U. (2023). PGT-M for Premature Ovarian Failure Related to CGG Repeat Expansion of the FMR1 Gene. Genes.

[51] ACOG (2010). Committee Opinion No. 469: Carrier screening for fragile X syndrome. Obstet. Gynecol..

[52] Welt C.K., Smith P.C., Taylor A.E. (2004). Evidence of early ovarian aging in fragile X premutation carriers. J. Clin. Endocrinol. Metab..

[53] Rohr J., Allen E.G., Charen K., Giles J., He W., Dominguez C., Sherman S.L. (2008). Anti-Mullerian hormone indicates early ovarian decline in fragile X mental retardation (FMR1) premutation carriers: A preliminary study. Hum. Reprod..

[54] Rajendra K., Bringman J.J., Ward J., Phillips O.P. (2008). Who should be tested for fragile X carriership? A review of 1 center’s pedigrees. Am. J. Obstet. Gynecol..

[55] Castilla J.A., Abellán F., Alamá P., Aura M., Bassas L., Clúa E., Guillén J., Manau D., Rueda J., Ruiz M. (2020). Genetic screening in gamete donation: Recommendations from SEF, ASESA, AEBM-ML, ASEBIR and AEGH. Med. Reprod. Embriol. Clínica.

[56] Practice Committee of the American Society for Reproductive Medicine and the Practice Committee for the Society for Assisted Reproductive Technology (2021). Guidance regarding gamete and embryo donation. Fertil. Steril..

[57] Dondorp W., De Wert G., Pennings G., Shenfield F., Devroey P., Tarlatzis B., Barri P., Diedrich K., Eichenlaub-Ritter U., Tuttelmann F. (2014). ESHRE Task Force on Ethics and Law 21: Genetic screening of gamete donors: Ethical issues. Hum. Reprod..

